# Supplementary material for: A Five Glutamine-Associated Signature Predicts Prognosis of Prostate Cancer and Links Glutamine Metabolism with Tumor Microenvironment
Source: J Clin Med. 2023 Mar 14;12(6):2243. doi: 10.3390/jcm12062243 (PMC10056698; doi:10.3390/jcm12062243)
Supplement: Supplementary file 1 [file jcm-12-02243-s001.zip › jcm-2262092-supplementary.pdf]

Supplementary information

Supplementary Figures

Figure S1

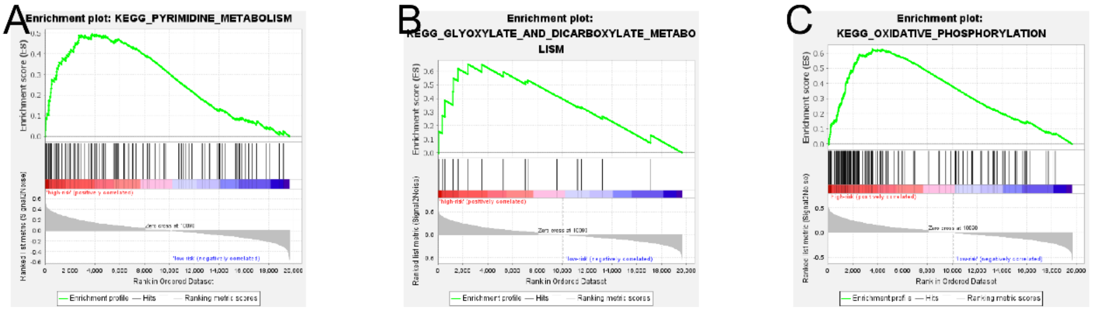

Figure S2

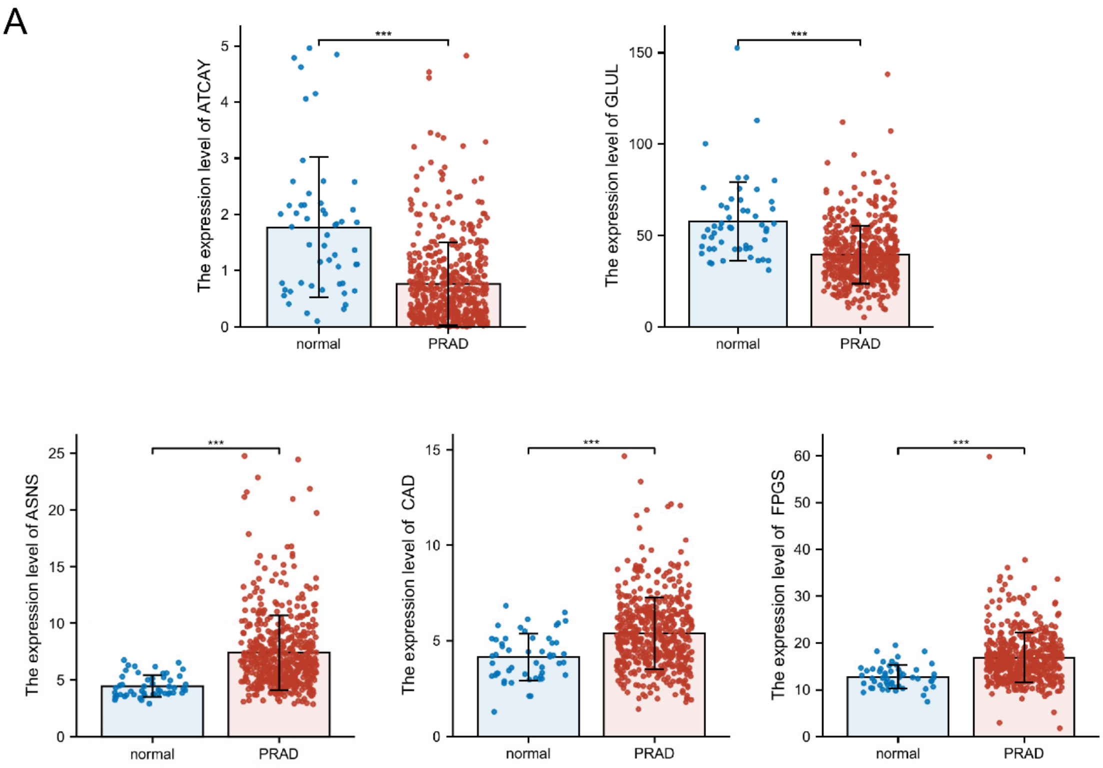

Supplementary Figure Legends

Figures S1. Mutational landscape and single sample GSEA analysis in high and low-risk groups of prostate cancer

(A-C) Pyrimidine metabolism,Glyoxylate and dicarboxylate metabolism and Oxidative phosphorylation in the high risk group

Figures S2. Exprerimental validation 5 key genes expression levels in PCa clinical samples

(A) The expression levels of ATCAY, GLUL, ASNS, CAD, FPGS in TCGA database

Supplementary Tables

Table S1. The forward and reverse primers of 5 key genes and GAPDH

**ATCAY**	
Forward primer	TGGCCCCAGAGATCAACATT
Reverse primer	TCGTCTTCCCACTCCAGTTC
**GLUL**	
Forward primer	GTGTGTGGAAGAGTTGCCTG
Reverse primer	CTGCAGGCCTTCGATTGTAC
**ASNS**	
Forward primer	TTCTTCGTGTTGGATGGGGA
Reverse primer	GGAGAGGGCTGTGAGTTCTT
**CAD**	
Forward primer	ACAGGACCCTTCAATCTGCA
Reverse primer	ACTGAGGCACCTTTACTCCC
**FPGS**	
Forward primer	GACCCTCAGACACAGTTGGA
Reverse primer	ATCCCGTCTTCAGGCCATAG
**GAPDH**	
Forward primer	GTATCGTGGAAGGACTCATGAC
Reverse primer	ACCACCTTCTTGATGTCATCAT

Table S2. siRNA sequences of knockdown GLUL and ASNS

Cell Lines	Gene ID	sense（5'-3'）	antisense（5'-3'）
22Rv1	GLUL-Homo-1549	GCACGUGUCUUCUCAAUGATT	UCAUUGAGAAGACACGUGCTT
	ASNS-Homo-1021	GGCAGAUCAUAUUGGAAGUTT	ACUUCCAAUAUGAUCUGCCTT
DU145	GLUL-Homo-1549	GCACGUGUCUUCUCAAUGATT	UCAUUGAGAAGACACGUGCTT
	ASNS-Homo-517	CCUGGGUAGAGAUACAUAUTT	AUAUGUAUCUCUACCCAGGTT

Table S3.91 glutamine related genes from GSEA database

Gene name:

AADAT	ART4	CLN3	FTCD	GLUD1	MECP2	OAT
ADHFE1	ASL	CPS1	GAD1	GLUD2	MIR21	OTC
AGMAT	ASNS	CTPS1	GAD2	GLUL	MTHFS	PFAS
ALDH18A1	ASNSD1	CTPS2	GCLC	GLYATL1	NAGS	PHGDH
ALDH4A1	ASRGL1	DAO	GCLM	GLYATL1B	NIT2	PPAT
ALDH5A1	ASS1	DDAH1	GFPT1	GMPS	NOS1	PRODH
AMDHD1	ATCAY	DDAH2	GFPT2	GOT1	NOS2	PRODH2
ARG1	ATP2B4	DGLUCY	GGT1	GOT2	NOS3	PYCR1
ARG2	BLOC1S6	FAH	GLS	HAL	NOXRED1	PYCR2
ARHGAP11B	CAD	FPGS	GLS2	LGSN	NR1H4	PYCR3
COX16	COX5A	MDH1	NDUFB10	NDUFC2	PDP1	PNPO
SLC25A13	TALDO1	TNFRSF11B	KYAT1	RIMKLA	RIMKLB	SIRT4

SLC25A12	SLC39A8	SLC7A11	SLC7A7	TAT	UROC1	COX10
